# Case report: Cholecystoduodenostomy for cholestatic liver disease in a premature infant with cystic fibrosis and short gut syndrome

**DOI:** 10.1186/s12887-019-1443-5

**Published:** 2019-03-11

**Authors:** Laura K. Fawcett, John Widger, Guy M. Henry, Chee Y. Ooi

**Affiliations:** 1 Department of Respiratory Medicine, Sydney Children’s Hospital, level 0 South West Wing, High St, Randwick, NSW 2031 Australia; 2 Discipline of Paediatrics, School of Women’s and Children’s Health, Medicine, University of New South Wales, Sydney, NSW Australia; 3 Molecular and Integrative Cystic Fibrosis (miCF) Research Centre, Sydney Children’s Hospital, High Street, Randwick, NSW Australia; 4 Department of General Surgery, Sydney Children’s Hospital, High Street, Randwick, NSW Australia; 5 Department of Gastroenterology, Sydney Children’s Hospital, High Street, Randwick, NSW Australia

**Keywords:** Cystic fibrosis, Conjugated hyperbilirubinaemia, Prematurity, Short gut syndrome, Cholestasis

## Abstract

**Background:**

Cholecystoduodenostomy is a surgical procedure that bypasses the extrahepatic biliary tree and connects the gallbladder directly to the duodenum. This case describes the successful use of this procedure in a novel situation.

**Case presentation:**

A premature (34 weeks gestation) female infant with cystic fibrosis required a laparotomy on day 1 of life due to an intrauterine small bowel perforation. Resection of small bowel and ileostomy formation resulted in short gut syndrome, with 82 cm residual small bowel and intact ileocaecal valve. Post-ileostomy reversal at 2 months old, she developed conjugated hyperbilirubinaemia. Despite conservative management including increased enteral feeding, ursodeoxycholic acid, cholecystostomy drain insertion and flushes, her cholestatic jaundice persisted. A liver biopsy revealed an “obstructive/cholestatic” picture with fibrosis. To avoid further shortening her gut with an hepatoportoenterostomy, cholecystoduodenostomy was performed at 3 months of age with subsequent post-operative improvement and eventual normalisation of her clinical jaundice and liver biochemistry.

**Conclusions:**

This is the first reported case of a cholecystoduodenostomy being used successfully to treat an infant with persistent conjugated hyperbilirubinemia, cystic fibrosis and short gut syndrome. Cholecystoduodenostomy is a treatment option that with further study, may be considered for obstruction of the common bile duct in patients with short gut and/or where a shorter operating time with minimal intervention is preferred.

## Background

Cystic fibrosis (CF) is an autosomal recessive disease of inflammation, obstruction and infection caused by mutations in the CF transmembrane conductance regulator gene. Mutations in this gene lead to an abnormality of, or reduction in, Chloride channels in the apical membranes of cells present in a variety of organs including the lung, pancreas and liver. The chloride channel abnormality results in thick sticky mucous in the airways and pancreas [1]*.* Neonatal cholestasis and meconium ileus are recognised complications [1–4]. Obstruction of the biliary tract (intra and extrahepatic) occurs in cystic fibrosis due to inspissated bile. Hepatocyte damage is thought to result from the subsequent accumulation of toxic bile acids and depletion of hepatic antioxidants [2, 4]. Extrahepatic biliary tract obstruction can be overcome with bypass surgery or drainage procedures. The Kasai procedure for infants with biliary atresia is a common example of bypass surgery. Cholecystostomy, where a drain is inserted into the gallbladder to allow bile to drain externally overcomes the acute obstruction but is not a definitive procedure. Cholecystoduodenostomy is a less frequently used surgical procedure that bypasses the extrahepatic biliary tree and connects the gallbladder directly to the duodenum. Here we report a rare and unusual case which required an infrequently used intervention. We discuss the challenges in managing this infant and the successful use of cholecystoduodenostomy to bypass the biliary obstruction.

## Case presentation

**Fig. 1:**
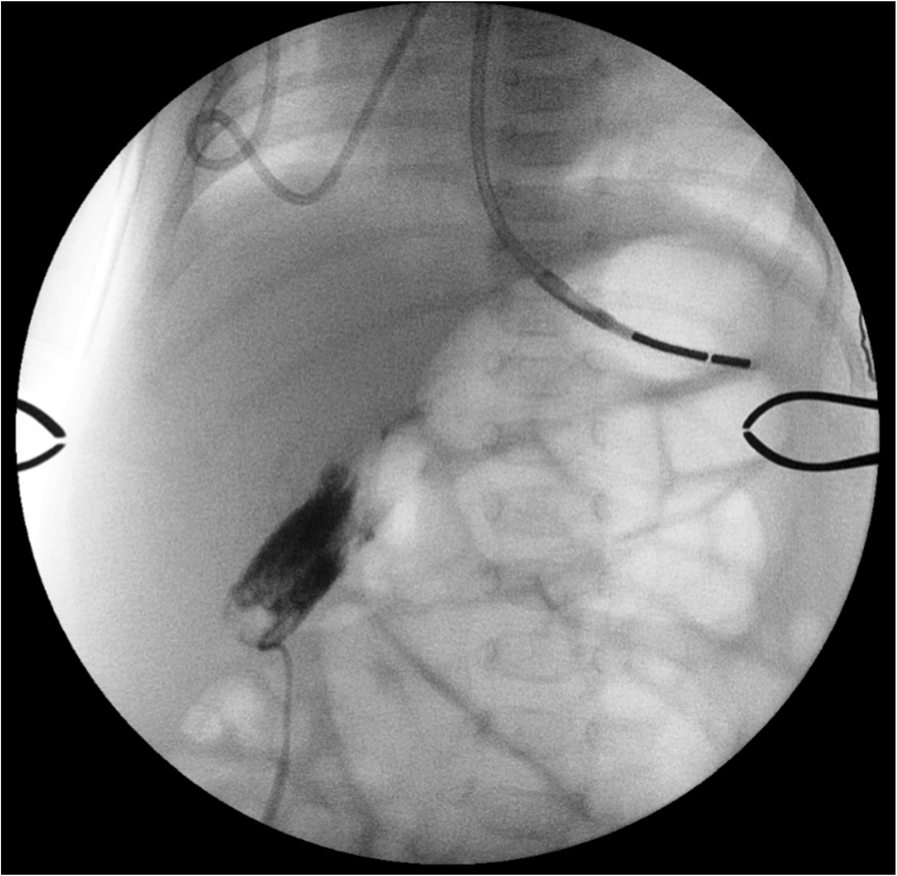
Intraoperative Cholangiogram demonstrating no filling of the biliary tree at 13 weeks of age

**Fig. 2:**
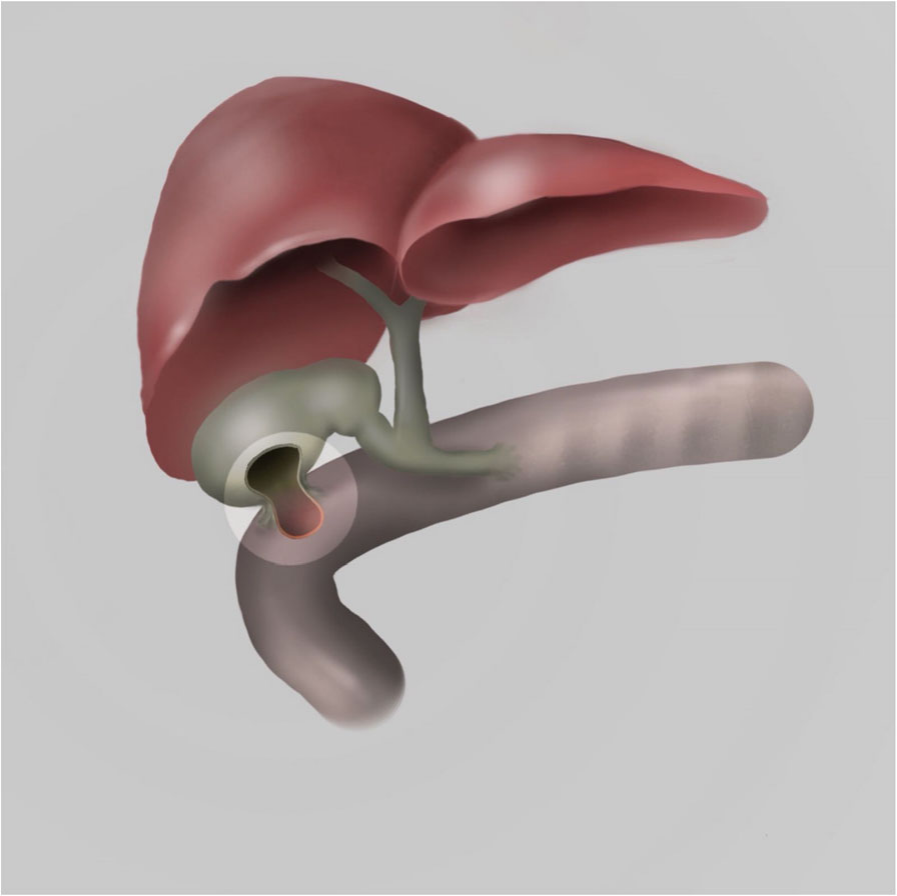
Cholecystoduodenostomy

**Fig. 3:**
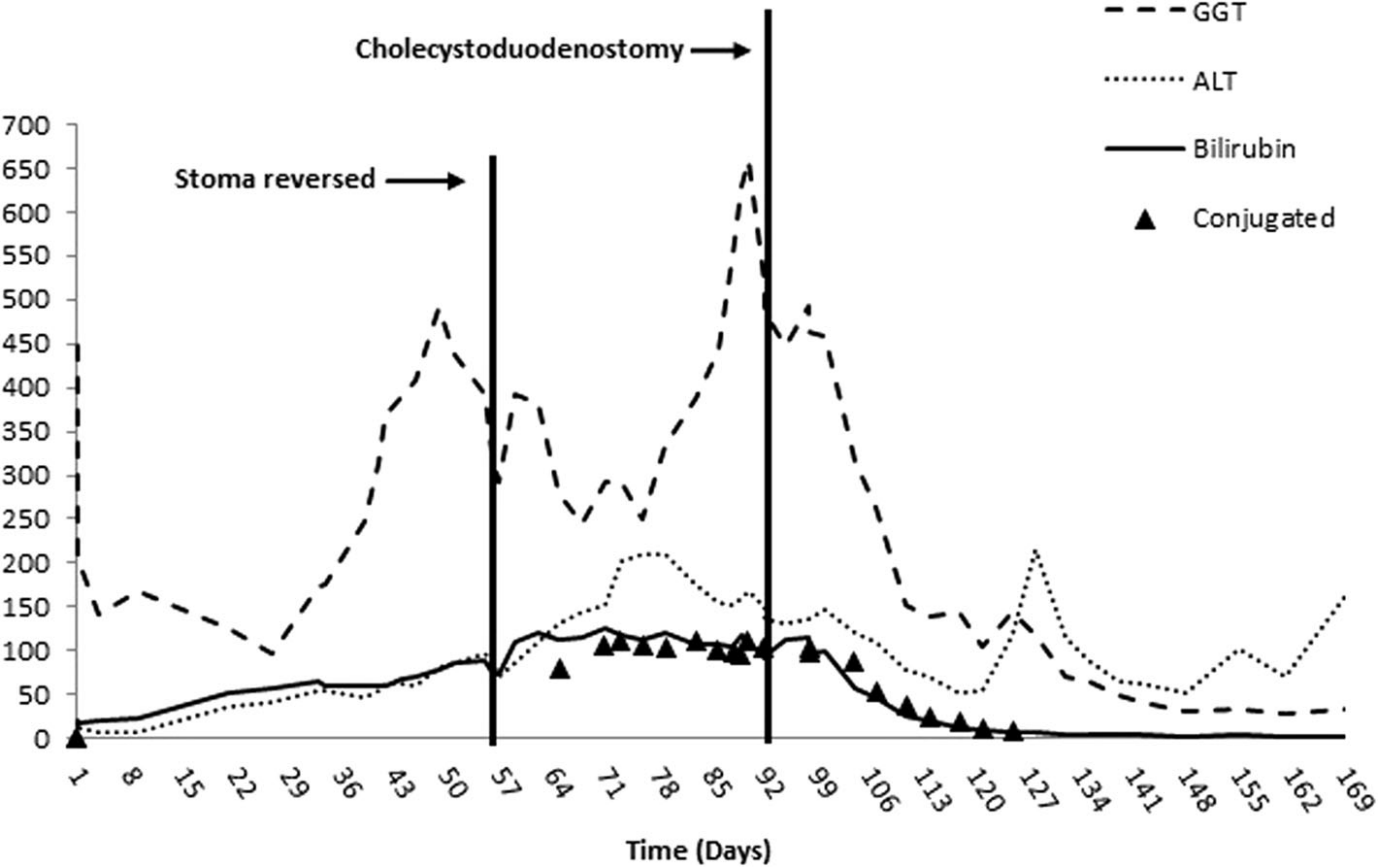
Graph of LFTs over time

A 2.28 kg female infant born at 33 weeks gestational age with antenatally diagnosed bowel obstruction and Cystic Fibrosis (F508Del/F508Del), underwent laparotomy with small bowel resection (82 cm residual small bowel and intact ileocaecal valve) and ileostomy formation on day 1 of life. Severe meconium peritonitis was present secondary to a small bowel perforation caused by meconium ileus and midgut volvulus. The ileostomy was reversed at 2 months of age. During this time she was dependent on parenteral nutrition as minimal enteral feeds were tolerated. Worsening jaundice (conjugated hyperbilirubinaemia) and acholic stools developed after the first month of age. These peaked at 2–3 months of age (total bilirubin 111 μmol/L, conjugated bilirubin 104 μmol/L, GGT U/L 660 U/L, ALT 167 U/L) despite treatment with ursodeoxycholic acid, cycling of parenteral nutrition and efforts to optimise enteral feeding. Hepatobiliary (HIDA) scans demonstrated no biliary drainage. During an open cholangiogram via the gallbladder (Fig. 1), contrast did not pass beyond the cystic duct. Liver biopsy taken at this time demonstrated fibrous expansion of portal tracts, cholestasis and bile duct proliferation. Differential diagnoses included inspissated bile due to CF, parenteral nutrition-related liver disease and biliary atresia. Daily flushing with normal saline and N-acetylcysteine via cholecystostomy and subsequent percutaneous cholangiograms were unsuccessful. Glucagon therapy was trialled following review of a case report, it was ceased due to adverse side effects [5]. Following extensive discussion between the patients’ parents, her CF team and surgeons, at 3 months of age, the patient underwent a cholecystoduodenostomy (Fig. 2) in order to bypass the extrahepatic biliary tract whilst preserving the infant’s biliary tract and her remaining small bowel. At the time of surgery, cannulation of the cystic duct was attempted. The cystic duct was punctured and the cannula flushed with saline but there was leakage. An intraoperative cholangiogram demonstrated ongoing leakage outside of the duct with subperitoneal tracking of contrast. A cholecystoduodenostomy was then performed as well as a repeat liver biopsy. The biopsy showed liver disease with minimal early nodular fibrotic changes. Her stool became formed and pigmented within 5 days. Bilirubin levels normalised post-operatively and her jaundice resolved within 1 month of surgery (Fig. 3). She was discharged home on full enteral feeds at 6 months of age. At 11 months of age the patient was admitted electively for insertion of gastrostomy due to ongoing requirement for overnight enteral feeds. She has introduced solids to her diet and her weight has continued to improve. At 18 months of age her BMI is now on the 37th centile. She has had no episodes of acute cholangitis and no elevated bilirubin levels. The patient continues on Trimethoprim/Sulphamethoxazole for *Staphylococcus aureus* prophylaxis (s.aureus prophlyaxis is standard care for CF patients under 2 years at SCH).

## Discussion and conclusions

**Fig. 4:**
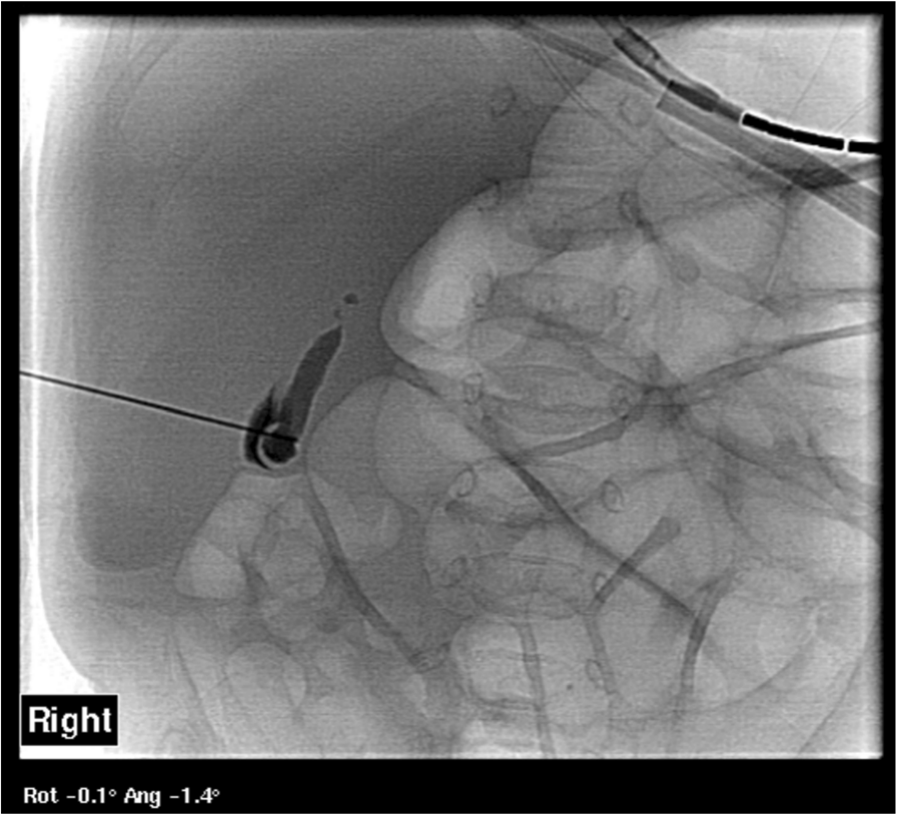
Percutaneous Cholangiogram demonstrating lack of filling of the biliary tree at 10 weeks of age

Our patient had intertwined issues of worsening liver disease (with concerns of future risk of cirrhosis), short gut syndrome and prematurity. Neonatal cholestasis is a rare manifestation of CF and can mimic biliary atresia and parenteral nutrition-related cholestasis [2]. Neonatal cholestasis due to CF usually resolves spontaneously [3]. Our case demonstrates the severe end of the spectrum of neonatal cholestasis with fibrosis evident on liver biopsy at 2 months of age, worsening liver biochemistry and jaundice despite medical management and subsequent insertion of a cholecystostomy drain. Definitive diagnosis of the problem was difficult due to the complexity of the case. Investigations to exclude biliary atresia were unable to do so, persistently showing a lack of filling of the extrahepatic biliary tract and no drainage of bile into the gut (Figs. 1 and 4). Biliary atresia has been reported in CF [6]. Optimal outcomes for any obstructive liver disease are related to timely surgery to bypass the obstruction. Various aforementioned therapies were unsuccessful in this patient. Our patient’s gastrointestinal issues were further complicated by intestinal failure caused by multiple factors including short gut syndrome and malabsorption due to CF and biliary insufficiency. Our patient required ongoing treatment with parenteral nutrition as only minimal enteral nutrition was tolerated and there was failure to thrive.

Hepatoportoenterostomy (Kasai procedure) is the preferred treatment for biliary atresia and may be used to bypass other causes of cholestasis due to bile duct obstruction [7]. However, this procedure requires mobilisation of a portion of the small bowel and would lead to further shortening of the gut. It involves removal of the extrahepatic biliary tract and the formation of a Roux-en-Y loop. We were concerned that the consequences of further shortening the gut would result in a longer period of dependence on parenteral nutrition, which would impact further on an already fibrotic liver. Portoduodenostomy is another biliary bypass procedure which does not require removal of any further small bowel. In cases where this has used the ileocaecal appendix to bypass biliary atresia there is a high risk for stone formation and obstruction because bile can stagnate in the ileocecum [8]. When this procedure is compared with the Kasai, improvement of hyperbilirubinaemia is less successful, with the portoduodenostomy group having a higher number of patients proceeding to liver transplantation [9]. With the viscous bile which is present in CF, failure of biliary drainage could be hypothesised to be of increased risk in our patient’s case. Cholecystoduodenostomy is an alternative procedure that attaches the gallbladder to the duodenum, which leaves the small bowel and extrahepatic biliary tree intact. It is a less invasive and shorter operation than the Kasai. Complications of cholecystoduodenostomy include cholangitis [10] as in this reported case of the procedure being used to manage a case of extra hepatic biliary atresia. There may secondary benefit of antibiotic prophylaxis in our patient, reducing her risk of cholangitis.

There was an urgency to manage the obstruction in our case, which required surgical intervention following failure of conservative management. The patient was small (3.9 kg), had poor weight gain (contributed to by short gut and malabsorption due to CF), has had previous abdominal surgeries and had adhesions from her meconium peritonitis. To the best of our knowledge, this is the first reported case of an infant with CF and short gut syndrome successfully undergoing cholecystoduodenostomy for neonatal cholestasis from extrahepatic biliary obstruction. Cholecystoduodenostomy may be a surgical option for obstruction of the common bile duct in affected infants with short gut syndrome and/or other factors where a shorter, less invasive surgery is required. Further experience and evaluation of cholecystoduodenostomy in this patient population is recommended to determine its efficacy and long term outcomes.

## Abbreviations

CF: Cystic Fibrosis
LFT: Liver Function Test
